# Hyperintensity of Cerebrospinal Fluid on T2-Weighted Fluid-Attenuated Inversion Recovery Magnetic Resonance Imaging Caused by High Inspired Oxygen Fraction

**DOI:** 10.3389/fvets.2017.00219

**Published:** 2017-12-18

**Authors:** Melania Moioli, Olivier Levionnois, Veronika M. Stein, Gertraud Schüpbach, Marta Schmidhalter, Daniela Schweizer-Gorgas

**Affiliations:** ^1^Clinical Radiology, Department of Clinical Veterinary Medicine, Vetsuisse-Faculty, University of Bern, Bern, Switzerland; ^2^Clinical Anesthesiology, Department of Clinical Veterinary Medicine, Vetsuisse-Faculty, University of Bern, Bern, Switzerland; ^3^Clinical Neurology, Department of Clinical Veterinary Medicine, Vetsuisse-Faculty, University of Bern, Bern, Switzerland; ^4^Department of Clinical Research and Veterinary Public Health, Veterinary Public Health Institute, Vetsuisse-Faculty, University of Bern, Bern, Switzerland

**Keywords:** cerebrospinal fluid, oxygen, T2-weighted fluid-attenuated inversion recovery, magnetic resonance imaging, brain, dog, cat

## Abstract

In veterinary medicine, patients undergo magnetic resonance imaging (MRI) under general anesthesia to enable acquisition of artifact-free images. The fraction of inspired oxygen (FiO_2_) ranges between 30 and 95%. In humans, a high FiO_2_ is associated with incomplete signal suppression of peripheral cerebrospinal fluid (CSF) spaces on T2-weighted fluid-attenuated inversion recovery (T2w-FLAIR) sequences. The influence of FiO_2_ on T2w-FLAIR images remains unreported in small animals. The aim of this prospective study was to investigate whether a high FiO_2_ is associated with hyperintensity in peripheral CSF spaces on T2w-FLAIR images in dogs and cats. Client-owned patients undergoing brain MRI were prospectively enrolled. Animals with brain parenchymal abnormalities and/or meningeal contrast enhancement on MRI images and/or abnormal CSF analysis were excluded. Consequently, twelve patients were enrolled. Anesthesia was maintained by isoflurane 0.5–1 minimal alveolar concentration in 30% oxygen. After acquisition of transverse and dorsal T2w-FLAIR images, the FiO_2_ was increased to 95%. The T2w-FLAIR sequences were then repeated after 40 min. Arterial blood gas analysis was performed in six patients at the same time as T2w-FLAIR sequence acquisition. Plot profiles of the signal intensity (SI) from CSF spaces of three cerebral sulci and adjacent gray and white matter were generated. SI ratios of CSF space and white matter were compared between the T2w-FLAIR images with 30 and 95% FiO_2_. An observer blinded to the FiO_2_, subjectively evaluated the SI of peripheral CSF spaces on T2w-FLAIR images as high or low. There was significant difference in the partial pressure of oxygen between the two arterial samples (*P* < 0.001). The SI ratios obtained from the T2w-FLAIR images with 95% FiO_2_ were significantly higher compared with those obtained from the T2w-FLAIR images with 30% FiO_2_ (*P* < 0.05). The peripheral CSF spaces were subjectively considered hyperintense in 11 of 12 cases on T2w-FLAIR images with 95% FiO_2_ (*P* < 0.005). A clear difference in SI, dependent on the FiO_2_ was seen in the peripheral CSF spaces on T2w-FLAIR images. In conclusion, the influence of FiO_2_ must be considered when differentiating pathological and normal CSF spaces on T2w-FLAIR images in dogs and cats.

## Introduction

T2-weighted fluid-attenuated inversion recovery (T2w-FLAIR) sequence consists of an inversion recovery pulse to suppress the signal from cerebrospinal fluid (CSF) and a long echo time to produce a heavily T2-weighted sequence. The T2w-FLAIR sequence is an essential part of the magnetic resonance imaging (MRI) protocol of the brain for both human and veterinary medicine (1–10). Compared with conventional T2-weighted and proton density-weighted imaging, T2w-FLAIR sequence provides better detection and evaluation of lesions within or adjacent to the CSF and higher lesion conspicuity within the brain parenchyma (3, 6). The term “hyperintense cerebrospinal fluid” is used to describe failed suppression of CSF signal on T2w-FLAIR images of the brain. Incomplete suppression of the CSF signal on T2w-FLAIR images is seen with subtle variations in the composition of CSF due to pathologic conditions such as subarachnoid hemorrhage, infarction, meningitis, vascular diseases, and neoplastic conditions (6, 9, 11, 12). However, hyperintense CSF on T2w-FLAIR images can be detected without a definite CSF abnormality. Several MRI artifacts lead to incomplete suppression of CSF signal on T2w-FLAIR images such as chemical shift, truncation, cross-talk, or overlapping of imaging planes (13). Patient motion, CSF flow, inhomogeneity in the amplitude of the inversion pulse, suboptimal inversion time, or magnetic field inhomogeneity can also lead to hyperintense signal of CSF on T2w-FLAIR (13). Magnetic field inhomogeneity can be caused by paramagnetic substances (14). Oxygen is a paramagnetic substance with two unpaired electrons, it reduces T1-relaxation time and causes high signal intensity (SI) of CSF on T2w-FLAIR images in patients receiving oxygen supplementation (2, 6, 9, 11, 13–18).

The major exchange of oxygen between arterial blood and the CSF takes place at the choroid plexus of the ventricles. Arterial partial pressure of oxygen (PaO_2_) is a measurement of the pressure of dissolved oxygen in the arterial blood. It is predominantly influenced by how much oxygen diffuses from the airspace of the lungs into the blood. A higher arterial partial pressure of oxygen leads to increased diffusion of the oxygen into the CSF according to the pressure gradient. Under physiological condition, the arterial partial pressure of oxygen is proportional to the inspired oxygen fraction (FiO_2_). When breathing room air, the FiO_2_ is approximately 21% and increases up to 100% when oxygen is supplemented. In humans, oxygen supplementation with a FiO_2_ above 60% influences CSF signal on T2w-FLAIR images (11, 13, 16).

In veterinary medicine, patients undergo MRI under general anesthesia and oxygen supplementation is generally provided at various FiO_2_, often up to 100% (19). However, the influence of oxygen supplementation on CSF SI has been neglected. Therefore, the aim of this prospective study was to investigate the influence of oxygen supplementation on SI of CSF on T2w-FLAIR sequences in dogs and cats.

## Materials and Methods

Client-owned dogs and cats undergoing MRI of the brain presented to the Small Animal Clinic of the Vetsuisse-Faculty, University of Bern, were prospectively enrolled between June 2016 and May 2017. The study was performed in agreement with the local ethical regulations (Veterinary Office, Canton of Bern, Switzerland—BE53/16 and No. 27510) with signed owner consent.

All dogs and cats underwent clinical and neurological examination, followed by preanesthetic bloodwork, which included hematology and biochemistry. Patients were only included if they were deemed to be cardiovascularly stable and did not require preoxygenation prior to induction of general anesthesia.

Each patient was premedicated with either a combination of acepromazine (0.005–0.02 mg/kg) and methadone (0.1–0.4 mg/kg) or methadone alone (0.1–0.4 mg/kg), administered intravenously or intramuscularly. In two patients, dexmedetomidine (0.001–0.01 mg/kg) was additionally administered to maintain cooperative sedation. Induction of general anesthesia was performed with propofol (2–4 mg/kg) administered intravenously to allow for endotracheal intubation. General anesthesia was maintained with isoflurane (0.5–1.5%) in 30% oxygen to target a PaO_2_ of approximately 150 mmHg (under usual clinical conditions). The FiO_2_ was continuously monitored by a calibrated gas analyzer (Datex Ohmeda S5 Anesthesia Monitor, GE Healthcare) from a side-stream sampling line at the end of the endotracheal tube. Adjustments were made manually with two flowmeters providing air and oxygen, respectively. After the first two sequences, the FiO_2_ was increased to at least 95% to target a PaO_2_ of approximately 600 mmHg, and maintained until the end of the MRI examination. During MRI, the patients were mechanically ventilated and the ventilation parameters adjusted in order to maintain normocapnia (Et CO_2_ 35–45 mmHg). Continuous monitoring of peripheral oxygen saturation (SpO_2_) by tongue pulse oxymetry, respiratory spirometry, and breathing gas analysis including inspired and expired O_2_, CO_2_, and anesthetic inhalant were provided. Core temperature was measured *via* an esophageal probe. Plasma A-Lyte^®^ (Baxter AG) was infused at a rate of 5 mL/kg/h. Hypotension, defined as a mean blood pressure lower than 70 mmHg, was initially treated with a crystalloid bolus (10 mL/kg infused over 10 min). Non-responsive hypotension was then treated with a colloid bolus (Voluven 2 mL/kg delivered IV over 10 min) and finally with dopamine infusion starting with an infusion rate of 5 mcg/kg/min. The metatarsal artery was catheterized in a group of patients (weight >6 kg) and two arterial blood samples collected at the time of T2w-FLAIR sequence acquisition.

The animals were examined in a high-field MRI scanner [Philips Panorama HFO 1.0 T (Philips Medical Systems Nederland B.V., Best, the Netherlands)]. First, T2w-FLAIR sequences in transverse and dorsal planes were acquired (echo time 140 ms, repetition time 11,000 ms, inversion time 2,600 ms, slice thickness 3–3.5 mm, interslice gap 0.5 mm). After increasing FiO_2_, T2-weighted FSE sequence in sagittal and transverse planes, T1-weighted FSE in dorsal and transverse planes, T2* in transverse plane, and DWI in transverse plane were acquired.

T2-weighted fluid-attenuated inversion recovery sequences were repeated 40 min after the FiO_2_ increase. Afterward, Gadodiamide (0.5 mmol/mL, Omniscan, GE Healthcare, Germany) was administered at a dose of 0.15 mmol/kg intravenously and T1-weighted spin-echo sequence with fat suppression in the transverse plane and T1-weighted spin-echo in the dorsal plane were acquired.

After completion of the MRI examination, a CSF sample was obtained from the cerebellomedullary cistern.

Patients were excluded if MRI showed brain parenchymal abnormalities and/or abnormal meningeal contrast enhancement and/or if the CSF was abnormal on routine analysis (a normal CSF analysis consisted of CSF cells count below 5 cells/μL and/or CSF protein below 25 mg/dL).

All the measurements were performed by a second year diagnostic imaging resident (Melania Moioli).

**Figure 1:**
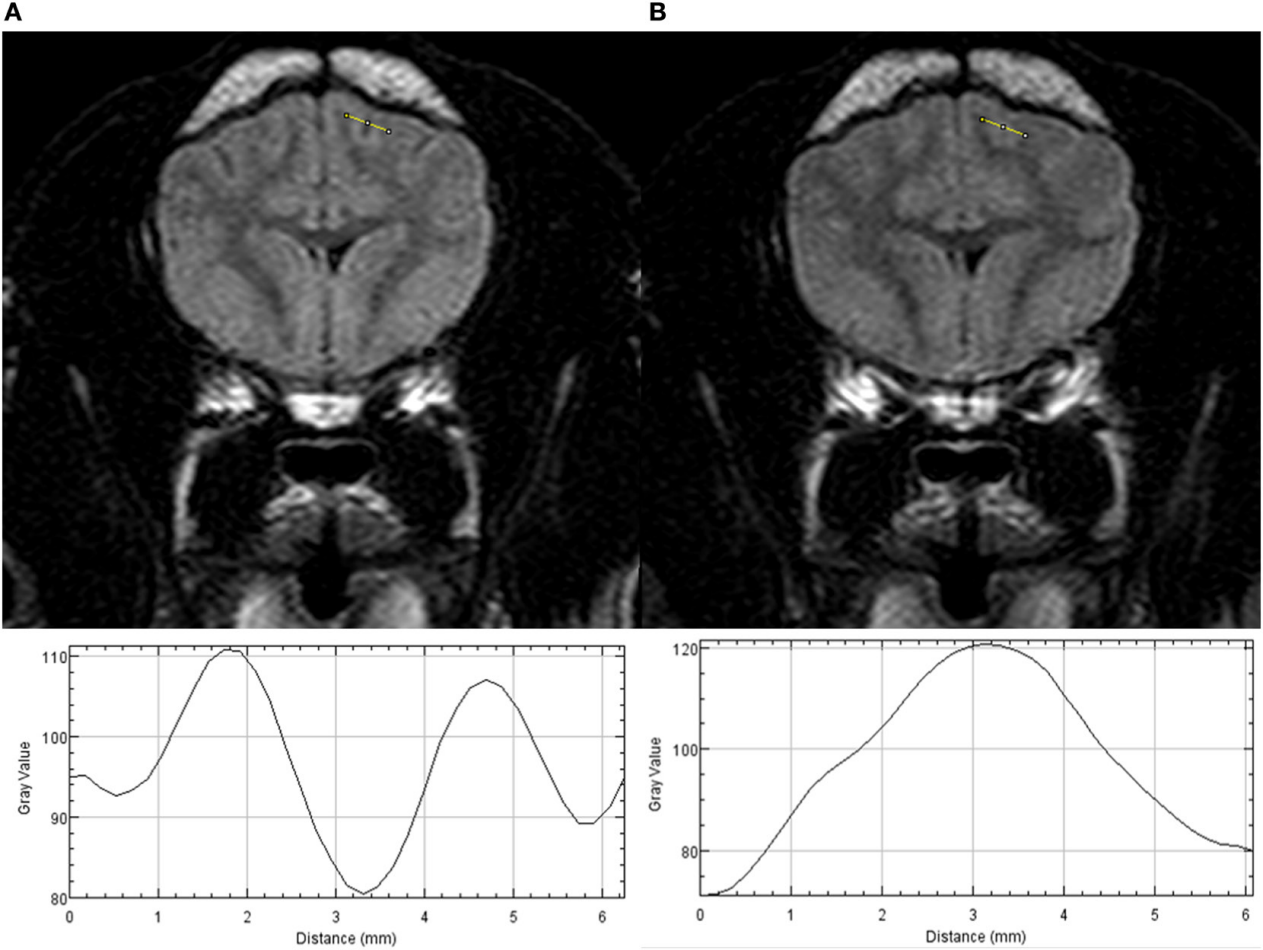
Transverse T2w-FLAIR images at the level of the forebrain in a 6-month-old male neutered Pug acquired with 30% FiO_2_ **(A)** and with 95% FiO_2_ **(B)**, are presented on the top. Plot profiles of the signal intensity (SI) of the cerebrospinal fluid (CSF) space within the marginal left sulcus and of the adjacent gray and white matter have been generated from both images and are presented in the lower part of the figure. Note the different SI values of the CSF space on the y axis the CSF space has the lowest SI value on the plot profile obtained from the T2w-FLAIR acquired with 30% FiO_2_ and the highest SI value on the plot profile obtained from the T2w-FLAIR acquired with 95% FiO_2_.

Plot profiles of the SI from CSF spaces of three cerebral sulci (two on transverse T2w-FLAIR images and one on dorsal T2w-FLAIR images) and adjacent gray and white matter from T2w-FLAIR images acquired with a FiO_2_ of 30% and 95% were generated using ImageJ^1^ (Figure 1). If possible, profiles of the marginal sulcus, ectomarginal sulcus, and caudal suprasylvian sulcus were generated. If these sulci were not visible, the endomarginal sulcus, precruciatus sulcus, presylvian sulcus, middle ectosylvian sulcus, cruciatus sulcus, or rostral sulcus were used. On each plot profile, the SI values for the CSF space and adjacent gray and white matter were recorded.

Standardized regions of interest encompassing the same area were set over the right and left lateral ventricles and the right and left thalamus ensuring they were in the same location on both the right and the left side and on both T2w-FLAIR images acquired with a FiO_2_ of 30 and 95%.

Signal intensity ratios were calculated from T2w-FLAIR images acquired with a FiO_2_ of 30 and 95%. The CSF space to white matter SI ratio and the gray matter to white matter SI ratio of three sulci and adjacent gyri were calculated. The lateral ventricle to thalamus SI ratio was calculated on the right and left side. The mean and SD of the ratios were calculated.

The ratios and the mean of the ratios from corresponding T2w-FLAIR images acquired with 30 and 95% FiO_2_ were compared: the CSF space to white matter ratio of every single sulcus and adjacent white matter, the white matter to gray matter ratio of every single gyrus adjacent to the sulcus, the mean of the CSF space to white matter ratios, the mean of the white matter to gray matter ratios, and the mean of the ventricle to thalamus ratios.

Subjective assessment of the corresponding T2w-FLAIR sequences was performed using a DICOM viewer (IMPAX EE R20 XVI SU1; Agfa HealthCare Gmbh, Germany). A board certified radiologist (Marta Schmidhalter) blinded to the FiO_2_ during acquisition of the T2w-FLAIR sequences evaluated whether the SI within peripheral CSF spaces showed complete or incomplete suppression. First, the observer was presented with single images in a random order at the level of forebrain, brainstem, and cervical spine from transverse T2w-FLAIR sequences obtained either with 30% FiO_2_ or with 95% FiO_2_, resulting in 72 evaluations. Second, the observer was presented with both transverse and dorsal images of the T2w-FLAIR sequences obtained with 30 or 95% FiO_2_, and she evaluated all peripheral CSF spaces, resulting in 24 evaluations. Third, the observer was presented with the pair of T2w-FLAIR sequences acquired with 30 and 95% FiO_2_ in both planes and compared the SI of the CSF spaces (12 evaluations).

The statistical analysis was performed by a second year diagnostic imaging resident (Melania Moioli) under supervision of a biostatistician (Gertraud Schüpbach). Quantitative data were assessed for normality using the Shapiro Wilk *W* test. All data were normally distributed, reported as mean ± SD and analyzed by a paired *T*-test. Subjective evaluation was analyzed using the McNemar test. All data were analyzed using a statistical software package [NCSS 10 Statistical Software (2015) NCSS, LLC., Kaysville, UT, USA^2^]. The level of significance was set to *P* ≤ 0.05.

## Results

Twenty-three patients were enrolled in the study. Eleven patients were excluded because of brain parenchymal abnormalities on MRI or the need for preoxygenation during anesthesia induction. Twelve patients (10 dogs and 2 cats) were therefore included in the study. One each of the following dog breeds were represented: Cavalier King Charles Spaniel, Chihuahua, Maltese, Great Dane, English Springer Spaniel, English Cocker Spaniel, Dalmatian, Flat Coated Retriever, Labrador Retriever, and one mixed breed dog. The gender of the dogs was equally distributed with five female (one intact, four spayed) and five male dogs (three intact, two neutered). The mean age of the dogs was 7.8 years (SD: 2.7, range: 2.5–12 years) and the mean bodyweight was 30.8 kg (SD 13.9, range: 2.4–53 kg). Cats included a male castrated Domestic Shorthair (11 years of age, bodyweight 5.45 kg) and one female Maine Coon (5 years of age, 5.5 kg). Eight patients presented due to seizures, two for behavioral changes, one for dropped jaw, and one for acute progressive tetraparesis.

In six dogs, an arterial catheter was placed in the metatarsal artery and arterial blood samples collected. The mean PaO_2_ was 140.33 mmHg (SD 35.51 mmHg, range 92.9–188.1 mmHg) with 30% FiO_2_ and 492.35 mmHg (SD 54.52 mmHg, range 431.2–566.7 mmHg) with 95% FiO_2_. The paired *T*-test showed a significant difference between the two means (*P* < 0.001).

Signal intensity ratios were calculated from T2w-FLAIR images acquired with 30 and 95% FiO_2_ and were normally distributed. The objective measurements are presented in Tables 1–3. There was a significant difference in the CSF to adjacent white matter SI ratio of the three sulci considered separately (*P* ≤ 0.05, Table 1) as well as in the mean of the CSF spaces to adjacent white matter ratios acquired with 30 and 95% FiO_2_ (*P* ≤ 0.001, Table 1). There was no significant difference in the gray to white matter SI ratio of the gray and white matter adjacent to the three sulci (Table 2) as well as no significant difference in the mean of the gray to white matter ratios acquired with 30 and 95% FiO_2_ (Table 2). Additionally, no significant difference was found in the mean of the right and left ventricle to thalamus ratio with 30% FiO_2_ and 95% FiO_2_ (Table 3).

**Table 1:** Values of the signal intensity (SI) ratios of CSF to adjacent white matter of three sulci and adjacent gyri and the mean ± SD are presented.

	1 CSF/WM		2 CSF/WM		3 CSF/WM		Mean (±SD) (1 + 2 + 3) CSF/WM	
Patient	30% FiO_2_	95% FiO_2_	30% FiO_2_	95% FiO_2_	30% FiO_2_	95% FiO_2_	30% FiO_2_	95% FiO_2_
1	1.66	1.17	1.15	1.59	1.14	1.45	1.31 (±0.3)	1.40 (±0.2)
2	1.42	1.52	0.94	1.03	1.19	1.33	1.18 (±0.2)	1.29 (±0.2)
3	0.89	1.24	1.18	1.43	1.21	1.47	1.10 (±0.2)	1.38 (±0.1)
4	1.22	1.28	1.24	1.49	1.14	1.26	1.20 (±0.1)	1.34 (±0.1)
5	0.73	1.04	0.70	1.20	0.79	1.21	0.74 (±0.1)	1.15 (±0.1)
6	1.07	1.58	1.33	1.88	1.59	1.84	1.33 (±0.3)	1.77 (±0.2)
7	1.48	1.34	1.34	1.34	1.67	1.95	1.50 (±0.2)	1.54 (±0.4)
8	1.20	1.67	1.31	1.67	1.56	1.74	1.36 (±0.2)	1.69 (±0.0)
9	1.40	1.61	1.51	1.86	1.29	1.91	1.40 (±0.1)	1.79 (±0.2)
10	1.39	1.66	1.92	1.88	1.90	1.98	1.73 (±0.3)	1.84 (±0.2)
11	1.70	1.87	1.45	1.83	1.48	1.94	1.54 (±0.2)	1.88 (±0.1)
12	1.19	1.85	1.42	2.14	1.58	2.94	1.40 (±0.2)	2.31 (±0.6)
*P*-value	0.03*		0.0004*		0.003*		0.001*	

*The SI ratios were calculated from T2w-FLAIR images acquired with 30 and 95% FiO_2_*.

Significant P-values are marked with a *.

*1, first sulcus and adjacent gyrus; 2, second sulcus and adjacent gyrus; 3, third sulcus and adjacent gyrus; CSF, cerebrospinal fluid; WM, white matter; FiO_2_, fraction of inspired oxygen*.

**Table 2:** Values of the signal intensity (SI) ratios of gray to white matter adjacent to three sulci and the mean ± SD are presented.

	1 GM/WM		2 GM/WM		3 GM/WM		Mean (±SD) (1 + 2 + 3) GM/WM	
Patient	30% FiO_2_	95% FiO_2_	30% FiO_2_	95% FiO_2_	30% FiO_2_	95% FiO_2_	30% FiO_2_	95% FiO_2_
1	1.39	1.16	1.26	1.59	1.18	1.24	1.28 (±0.1)	1.33 (±0.3)
2	1.57	1.46	1.30	1.04	1.37	1.30	1.42 (±0.1)	1.27 (±0.2)
3	1.20	1.25	1.29	1.25	1.58	1.51	1.36 (±0.2)	1.34 (±0.2)
4	1.36	1.10	1.45	1.25	1.22	1.22	1.34 (±0.1)	1.19 (±0.1)
5	1.23	1.03	1.26	1.31	1.18	1.05	1.22 (±0.0)	1.13 (±0.2)
6	1.18	1.19	1.43	1.85	1.75	1.76	1.46 (±0.3)	1.60 (±0.4)
7	1.23	1.12	1.40	1.19	1.45	1.28	1.36 (±0.1)	1.19 (±0.1)
8	1.18	1.32	1.23	1.37	1.44	1.29	1.28 (±0.1)	1.33 (±0.0)
9	1.35	1.37	1.37	1.41	1.20	1.54	1.31 (±0.1)	1.44 (±0.1)
10	1.41	1.29	1.65	1.58	1.92	1.57	1.66 (±0.3)	1.48 (±0.2)
11	1.51	1.60	1.12	1.48	1.47	1.67	1.37 (±0.2)	1.58 (±0.1)
12	1.19	1.39	1.41	1.72	1.34	1.81	1.31 (±0.1)	1.64 (±0.2)
*P*-value	0.32		0.31		0.87		0.79	

*The SI ratios were calculated from T2w-FLAIR images acquired with 30 and 95% FiO_2_*.

Significant P-values are marked with a *.

*1, gyrus adjacent to the first sulcus; 2, gyrus adjacent to the second sulcus; 3, gyrus adjacent to the third sulcus; GM, gray matter; WM, white matter; FiO_2_, fraction of inspired oxygen*.

**Table 3:** Values of the mean ± SD of the lateral ventricle to thalamus signal intensity (SI) ratios of the left and right side are presented.

	Mean (±SD) (R + L) LV/THA	
Patient	30% FiO_2_	95% FiO_2_
1	0.29 (±0.2)	0.44 (±0.1)
2	0.30 (±0.0)	0.20 (±0.0)
3	0.10 (±0.0)	0.10 (±0.0)
4	0.71 (±0.0)	0.65 (±0.1)
5	0.23 (±0.0)	0.12 (±0.0)
6	0.22 (±0.1)	0.45 (±0.2)
7	0.69 (±0.3)	0.78 (±0.1)
8	0.71 (±0.1)	0.70 (±0.1)
9	0.19 (±0.0)	0.21 (±0.1)
10	0.45 (±0.2)	0.54 (±0.4)
11	0.30 (±0.0)	0.22 (±0.0)
12	0.27 (±0.1)	0.29 (±0.0)
*P*-value	0.49	

*The SI ratios were calculated from T2w-FLAIR images acquired with 30 and 95% FiO_2_*.

Significant P-values are marked with a *.

*R, right; L, left; LV, lateral ventricle; THA, thalamus; FiO_2_, fraction of inspired oxygen*.

In the first subjective blinded evaluation on single images at the level of the forebrain as well as at the level of the brainstem, a hyperintensity of the peripheral CSF spaces was seen in 3/12 T2w-FLAIR images acquired with 30% FiO_2_ and in 10/12 images acquired with 95% FiO_2_ (*P* < 0.05). At the level of the cervical spine, the peripheral CSF space was considered hyperintense in 5/12 T2w-FLAIR images acquired with 30% FiO_2_ and in 8/12 T2w-FLAIR images acquired with 95% FiO_2_. These results were not statistically significant.

In the second subjective evaluation, all transverse and dorsal images of T2w-FLAIR sequences acquired with 30 or 95% FiO_2_ were taken into account. Peripheral CSF spaces were considered hyperintense in 4/12 cases acquired with 30% FiO_2_ and 11/12 cases acquired with 95% FiO_2_. The McNemar test showed a significant difference between these results (*P* < 0.05).

**Figure 2:**
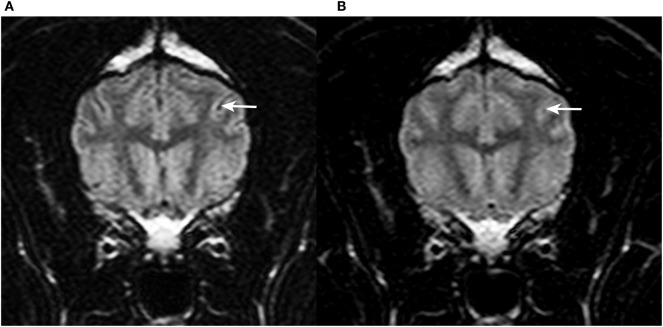
Transverse T2w-FLAIR images at the level of the forebrain in a 9-year-old female spayed Flat Coated Retriever acquired with 30% FiO_2_ **(A)** and with 95% FiO_2_ **(B)**, respectively. Note low signal intensity (SI) of peripheral cerebrospinal fluid (CSF) within cerebral sulci in **(A)** (arrow) and increase in SI of CSF within the same sulcus in **(B)** (arrow).

**Figure 3:**
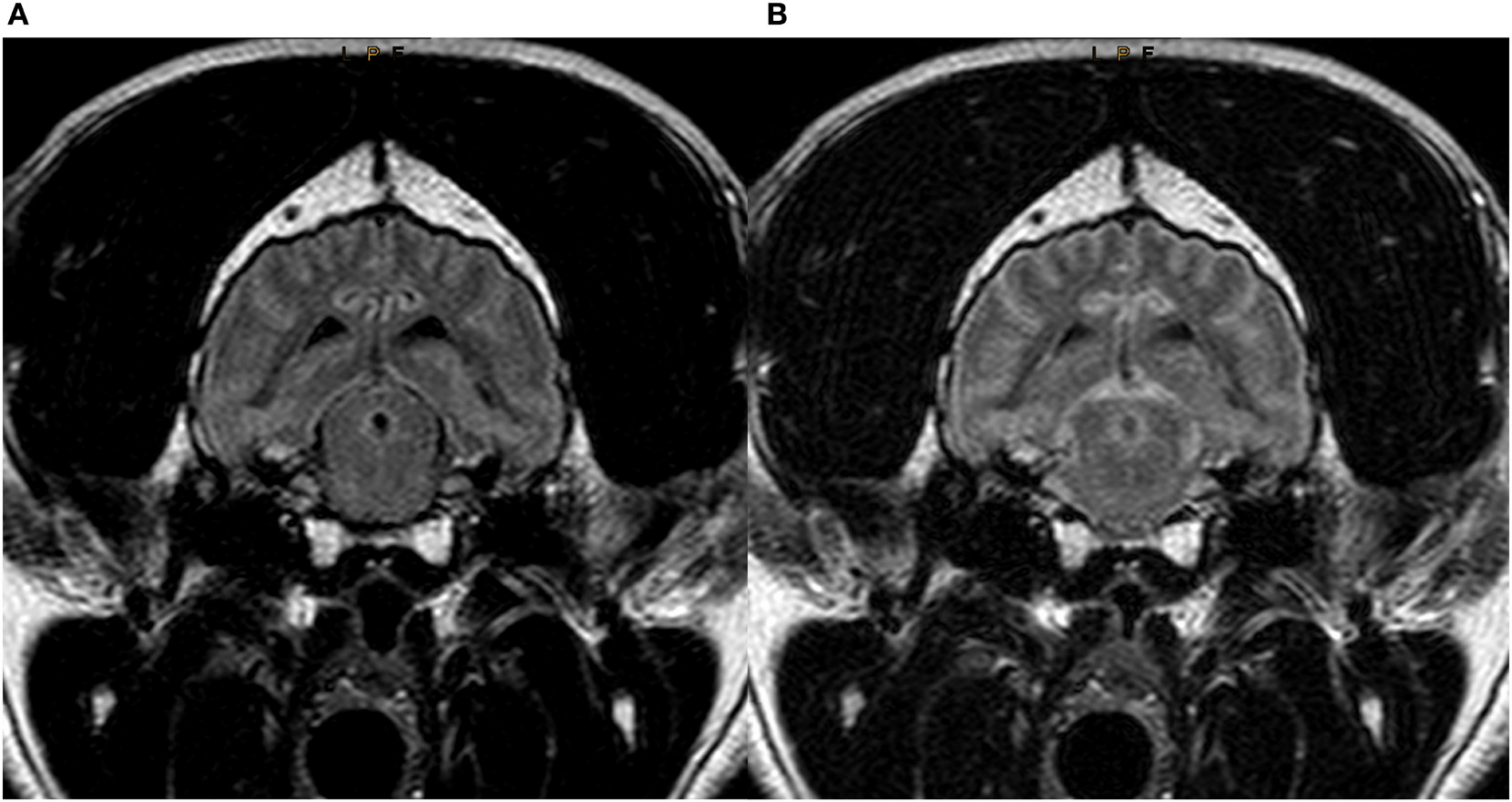
Transverse T2w-FLAIR images at the level of the brainstem in a 7-year-old male neutered Labrador Retriever acquired with 30% FiO_2_ **(A)** and with 95% FiO_2_ **(B)**, respectively. Note low signal intensity (SI) of peripheral cerebrospinal fluid (CSF) around the brainstem in **(A)** and increase in SI of CSF within the same region **(B)**.

**Figure 4:**
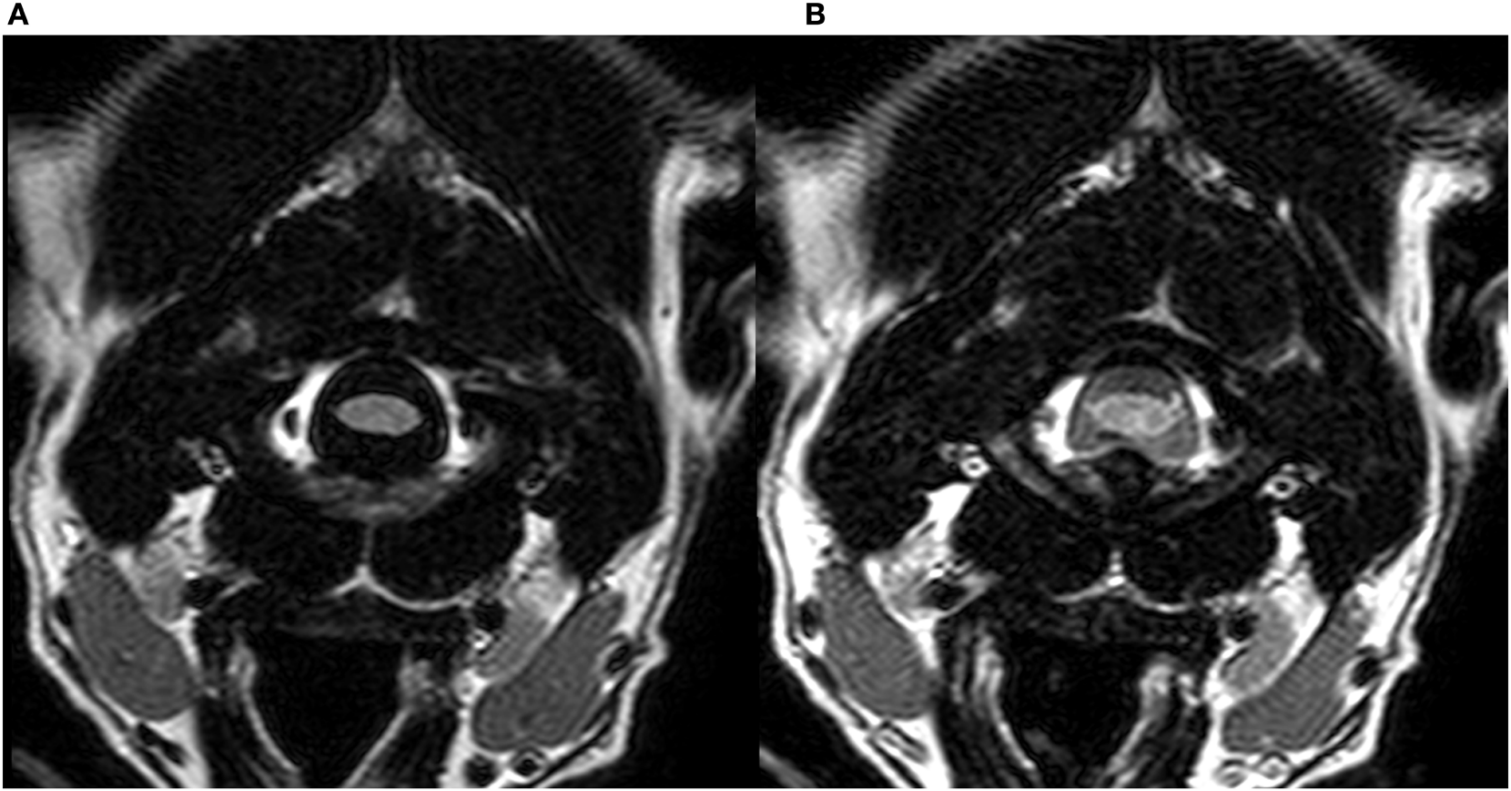
Transverse T2w-FLAIR images at the level of the cervical spine in a 3-year-old male neutered Dalmatian acquired with 30% FiO_2_ **(A)** and with 95% FiO_2_ **(B)**, respectively. Note low signal intensity (SI) of peripheral cerebrospinal fluid (CSF) around the cervical spinal cord in **(A)** and increase in SI of CSF within the same region **(B)**.

In the third evaluation, with direct comparison of the sequences acquired with 30 and 95% FiO_2_, the peripheral CSF spaces were considered hyperintense in 1/12 cases on images acquired with 30% FiO_2_ and in 11/12 cases on images acquired with 95% FiO_2_. The McNemar test showed significant difference between these results (*P* < 0.005). Transverse T2w-FLAIR images at the level of the forebrain, brainstem, and cervical spine acquired with 30% FiO_2_ and with 95% FiO_2_ are shown, respectively, in Figures 2–4.

## Discussion

“Hyperintensity of CSF spaces” is a term used to describe incomplete suppression of CSF signal on T2w-FLAIR imaging (11–13). It may indicate an altered CSF composition or meningeal disease as seen in meningoencephalitis, subarachnoid hemorrhage or neoplastic conditions (5, 6, 9, 11, 12, 20). In this study, hyperintensity of the peripheral CSF spaces was evident in patients with normal CSF analysis and no brain abnormalities on MRI, but it was associated with high FiO_2_ during the MRI examination. This agrees with observations in humans where CSF hyperintensity is associated with oxygen supplementation with a FiO_2_ higher than 60% during the T2w-FLAIR sequence acquisition (11, 13). In veterinary medicine, during general anesthesia, high FiO_2_ (up to 100%) is normally applied. This is because of its simplicity (only one gas supply) and because it is considered historically to be the safest method. However, lower FiO_2_ with oxygen-enriched air (FiO_2_ <50%) is sometimes preferred as it is sufficient to maintain arterial oxygenation and may reduce risk of oxygen toxicity. Indeed, lung aeration and gas exchange are improved with a FiO_2_ of 40% compared to FiO_2_ of 100% by reducing absorption atelectasis (19). In humans, it has been suggested, that a FiO_2_ less than 50% does not induce signal changes on T2w-FLAIR images due to insufficient free oxygen (13).

Hyperintensity of CSF spaces might easily be misinterpreted as pathologic conditions of the CSF space itself or the adjacent meninges. Meningeal diseases are hard to diagnose as T2w-FLAIR images, T1-weighted post-gadolinium, and subtraction images have a limited accuracy detecting lesions (20). Fat suppressed contrast-enhanced T1-weighted images with or without fluid-attenuated inversion recovery technique increase the conspicuity of contrast enhancing meningeal lesions (21, 22) but non-enhancing lesions are still difficult to detect.

As reported in humans, the present study showed incomplete suppression of CSF on T2w-FLAIR images in the peripheral CSF spaces such as the basilar cistern and the cerebral sulci, but not in the lateral ventricles (11, 13). This phenomenon may be explained by the diffusion of oxygen from the blood to the CSF along the pia-arachnoid surface of the brain, although the major exchange of oxygen between arterial blood and the CSF takes place at the choroid plexus of the ventricles (2, 11). A significant increase in oxygen partial pressure of CSF after 100% oxygen inhalation was detected using MRI as a non-invasive method to measure pO_2_ of human and fetal CSF (18). The highest oxygen concentrations were noted along the cortical surfaces, whereas lower concentrations were measured within the ventricular system (18). Furthermore, an increase in FiO_2_ leads to less dilution of dissolved oxygen in the peripheral CSF spaces due to their small volume compared to the lateral ventricles, leading to a stronger local paramagnetic effect (2).

The effect of paramagnetic substances is stronger with higher magnetic field strength (23, 24). We suspect that hyperintensity of CSF on T2w-FLAIR sequences is observed less often in low-field scanners. However, all studies investigating the influence of oxygen supplementation on CSF SI on T2w-FLAIR sequences, including the present one, were performed with high field scanners ranging from 1T to 3T (1, 2, 9–11, 15, 16, 25). Therefore, the impact on images acquired with low field scanners remains unknown.

The difference in SI of CSF spaces was not only evident when comparing ratios, but also noted on subjective evaluation. Differences between the T2w-FLAIR series with low and high FiO_2_ became more obvious on simultaneous comparison. Blind evaluation also revealed a significant difference. The evaluation of CSF at different levels of the brain by subjective evaluation and SI ratios showed both a significant difference between the low and high FiO_2_, while a smaller difference was found for the CSF surrounding the cervical spinal cord. CSF flow influences the SI of CSF and the high flow velocity at the level of the aqueduct and C1-C2 are possible causes for the inconsistent CSF signal. In humans, pulsation artifacts related to CSF flow are reported to occur in the basal, prepontine, and cerebellopontine angle cisterns and cause T2w-FLAIR hyperintensity in the subarachnoid space (6, 16). These artifacts are less common over the convexities of the cerebral hemispheres (6).

A limitation of this study was the heterogeneous group of animals, which included cats, small breed dogs, and even a Great Dane. They exhibited a large variety of skull shapes and brain morphology. In smaller animals, the sulci appeared smaller and distinction between the gyri and sulci as well as evaluation of CSF SI was more difficult. This meant that SI ratios could not be consistently performed at the same location. In addition to patient size, age plays a role in the appearance of sulci (26). The small sample size prohibited investigating the influence of size, body weight, skull morphology, and age on the appearance of CSF spaces (26, 27). Hyperintensity of the peripheral CSF spaces has also been reported to be associated with propofol anesthesia in one study (10). However, this is controversial as a different publication observed CSF hyperintensity with high FiO_2_ both with and without propofol anesthesia (11). In our study, all patients received propofol, but hyperintensity of the peripheral CSF spaces was only associated with high FiO_2_.

In conclusion, high FiO_2_ during MRI examination has been shown to result in hyperintense peripheral CSF spaces on T2w-FLAIR images in dogs and cats. In veterinary medicine where patients undergo general anesthesia during MRI examinations, this finding is of major importance for the evaluation of external CSF spaces and correct identification of pathological conditions.

## Ethics Statement

This prospective study was performed in agreement with the local ethical regulations (Veterinary Office, Canton of Bern, Switzerland—BE53/16 and No.27510). The animals included were patients presented to the Small Animal Clinic of the Vetsuisse-Faculty of Bern and undergoing MRI of the brain for reasons not related to the study. The patients were prospectively included if the owners provided signed informed consent. They were not designated “experimental animals.”

## Author Contributions

All authors contributed substantially to the conception or design of the study; or the acquisition, analysis, or interpretation of data. All authors drafted the work or revised it critically for important intellectual content and gave final approval before publication. All authors investigated and resolved questions relating to the accuracy or integrity of all part of the work.

## Conflict of Interest Statement

The authors declare that the research was conducted in the absence of any commercial or financial relationships that could be construed as a potential conflict of interest.

## Footnotes

^1^https://imagej.net.

^2^https://ncss.com/software/ncss.
